# Urinary levels of pro-fibrotic transglutaminase 2 (TG2) may help predict progression of chronic kidney disease

**DOI:** 10.1371/journal.pone.0262104

**Published:** 2022-01-18

**Authors:** Michelle Da Silva Lodge, Nick Pullen, Miguel Pereira, Timothy S. Johnson

**Affiliations:** 1 Academic Nephrology Unit and Sheffield Kidney Institute, University of Sheffield Medical School, Sheffield, United Kingdom; 2 Pfizer Global Research and Development, Cambridge, MA, United States of America; 3 Statistical Sciences and Innovation, UCB Pharma, Slough, United Kingdom; International University of Health and Welfare, School of Medicine, JAPAN

## Abstract

Renal clinical chemistry only detects kidney dysfunction after considerable damage has occurred and is imperfect in predicting long term outcomes. Consequently, more sensitive markers of early damage and better predictors of progression are being urgently sought, to better support clinical decisions and support shorter clinical trials. Transglutaminase 2 (TG2) is strongly implicated in the fibrotic remodeling that drives chronic kidney disease (CKD). We hypothesized that urinary TG2 and its ε-(γ-glutamyl)-lysine crosslink product could be useful biomarkers of kidney fibrosis and progression. Animal models: a rat 4-month 5/6^th^ subtotal nephrectomy model of CKD and a rat 8-month streptozotocin model of diabetic kidney disease had 24-hour collection of urine, made using a metabolic cage, at regular periods throughout disease development. Patients: Urine samples from patients with CKD (*n* = 290) and healthy volunteers (*n* = 33) were collected prospectively, and progression tracked for 3 years. An estimated glomerular filtration rate (eGFR) loss of 2–5 mL/min/year was considered progressive, with rapid progression defined as > 5 mL/min/year. Assays: TG2 was measured in human and rat urine samples by enzyme-linked immunosorbent assay (ELISA) and ε-(γ-glutamyl)-lysine by exhaustive proteolytic digestion and amino acid analysis. Urinary TG2 and ε-(γ-glutamyl)-lysine increased with the development of fibrosis in both animal model systems. Urinary TG2 was 41-fold higher in patients with CKD than HVs, with levels elevated 17-fold by CKD stage 2. The urinary TG2:creatinine ratio (UTCR) was 9 ng/mmol in HV compared with 114 ng/mmol in non-progressive CKD, 1244 ng/mmol in progressive CKD and 1898 ng/mmol in rapidly progressive CKD. Both urinary TG2 and ε-(γ-glutamyl)-lysine were significantly associated with speed of progression in univariate logistic regression models. In a multivariate model adjusted for urinary TG2, ε-(γ-glutamyl)-lysine, age, sex, urinary albumin:creatinine ratio (UACR), urinary protein:creatinine ratio (UPCR), and CKD stage, only TG2 remained statistically significant. Receiver operating characteristic (ROC) curve analysis determined an 86.4% accuracy of prediction of progression for UTCR compared with 73.5% for UACR. Urinary TG2 and ε-(γ-glutamyl)-lysine are increased in CKD. In this pilot investigation, UTCR was a better predictor of progression in patients with CKD than UACR. Larger studies are now warranted to fully evaluate UTCR value in predicting patient outcomes.

## Introduction

Proteinuria and albuminuria are the most frequently used predictors of renal function decline in kidney disease [1]. Changes in urinary albumin:creatinine ratio (UACR), as well as estimated glomerular filtration rate (eGFR), are commonly used as surrogate endpoints in chronic kidney disease (CKD) clinical trials [2, 3] and recognized as important risk factors for cardiovascular disease [4, 5]. Healthy individuals excrete < 150 mg/day protein or < 30 mg/day albumin. Microalbuminuria (30 to < 300 mg/day or 30–300 mg/L), typically measured as UACR (3–30 mg albumin/mmol creatinine), is often used as a gold standard for diagnosis of renal impairment and CKD development. Macroalbuminuria (300–3500 mg/day or UACR 30 to typically 9000 mg/mmol) is correspondingly used to estimate progression. However, albuminuria lacks specificity, being a common feature of aging, obesity, exercise, pregnancy, tobacco use, infections (e.g., human immunodeficiency virus [HIV], hepatitis C, and *Helicobacter pylori*) [6–11], and is also associated with vascular and inflammatory pathologies including inflammatory bowel disease, atherosclerosis, and periodontitis [12–14]. Microalbuminuria is often transitory and irresolute; thus, it is not a specific and reliable marker of CKD, especially in early disease.

While albuminuria is used clinically, there is a need for more reliable, sensitive, and accurate options. Several experimental biomarkers identified in blood or urine have been associated with CKD and its progression. These include markers of tubulointerstitial injury such as kidney injury molecule-1 (KIM-1) [15] and neutrophil gelatinase-associated lipocalin (NGAL) [16] as well as markers of glomerular injury such as podocin, podocalyxin, and nephrin [17–19]. Inflammatory markers have also been used to predict outcomes with chemokine (C-C motif) ligand 2 (CCL2), tumor necrosis factor (TNF)-like weak inducer of apoptosis, interleukin (IL)-18, C-reactive protein (CRP) [20–22] and, perhaps most notably, soluble TNF receptor 1/2 (TNFR1/2, members of the kidney risk inflammatory signature (KRIS) panel [23]). Other approaches have identified fibrosis associated proteins such as cadherin 11, macrophage mannose receptor C1, and phospholipid transfer protein [24] as possible predictors of progression. However, none of these markers have entered into common clinical use for a variety of reasons [25, 26], with the lack of predictive statistics showing a sufficient increase over conventional markers being a major factor [27–30].

Transglutaminase 2 (TG2) is a multifunctional protein associated with tissue stability, extracellular matrix (ECM) remodeling, apoptosis, cell adhesion, and wound healing [31, 32]. TG2 has been strongly implicated in kidney fibrosis by catalyzing the post-translational modification of ECM proteins with the formation of intramolecular ε-(γ-glutamyl)-lysine bonds [33]. TG2, ε-(γ-glutamyl)-lysine, collagens, and other ECM-related molecules can be detected in fibrotic kidney tissue from both animal models [33–36] and patients [37–40]. However, few studies have measured these markers and analyzed their clinical usability in urine or blood specimens.

Given the strong association of kidney TG2 and ε-(γ-glutamyl)-lysine crosslinking with experimental [33, 41] and human CKD [39, 42], coupled with the highly effective anti-fibrotic effects of TG2 inhibition observed in pre-clinical models [43, 44], we hypothesized that urine and circulating levels of TG2 and its crosslink product, ε-(γ-glutamyl)-lysine, may provide a novel non-invasive way of assessing the rate of fibrotic remodeling and thus CKD progression. To test this hypothesis, we assessed changes in urinary TG2 and ε-(γ-glutamyl)-lysine in two rat models of kidney fibrosis (5/6^th^ sub-total nephrectomy [SNx] and a streptozotocin [STZ]-induced diabetic nephropathy [DN] model on a uninephrectomy [UNx] background), allowing the tracking with disease development and direct correlation with renal histology. Subsequently, a prospective study involving 290 CKD patients and 33 healthy volunteers (HVs) measured urinary and serum TG2 and ε-(γ-glutamyl)-lysine and followed disease progression for 3 years. The predictive value of both molecules was assessed by receiver operating characteristic (ROC) curve analysis using urinary albumin:creatinine ratio (UACR) as a comparator.

## Materials and methods

### Models of experimental renal scarring

#### Experimental animals

All experiments were prospectively reviewed by the Animals in Science Regulation Unit of the UK Government Home Office and permissions granted by the secretary of state under project license: PPL 40/3660 in compliance with the Animal Scientific Procedures Act 1986.

Male Wistar Han rats (purchased from Harlan Laboratories, Bicester, UK) underwent 5/6^th^ SNx as a functional model of renal fibrosis, and STZ treatment with UNx as a model of DN, as previously described [43, 44]. In both models, animals were aged 8–10 weeks and weighed 200–250 g at the beginning of the experiment. Animals were weighed every other day and if a loss in body weight > 20% was observed, the animal was culled in line with PPL 40/3660. Rats were housed 2–4 animals per cage in 45% humidity on a 12/12 hour light-dark-cycle at 20–22°C and were allowed free access to rat chow (protein/casein content 18%; LabSure Ltd, Cambridge, UK) and water. Twenty-four-hour collection of urine was completed using a metabolic cage, at termination points throughout disease development. For SNx rats this was 7, 28 and 84 days and for STZ-DN rats was 1, 4 and 8 months, post-surgery. The animals were monitored daily for signs of distress, or symptoms consistent with the onset of end-stage renal failure and were culled if they exceeded pre-determined limits. Animals were sacrificed under deep anesthesia by exsanguination; a total of *n* = 32 for the SNx, and *n* = 39 for the STZ experiments. Five animals were culled when reaching the humane endpoints listed above (SNx *n* = 2; STZ *n* = 3).

#### 5/6^th^ subtotal nephrectomy

SNx was conducted on male Wistar Han rats by excision of 5/6^th^ of the kidney mass [44]. Two-thirds of the right kidney was resected following ligation (3/0 mersilk, Southern Syringe Services, London, UK) and excision of its lower and upper poles. Seven days later the contralateral kidney was resected. This procedure leads to the development of progressive glomerulosclerosis and tubule-interstitial fibrosis over 3 months. Control rats had dermal incisions without manipulation of the kidney. Rats (total *n* = 30) were sacrificed at 7 (sham *n* = 4; SNx *n* = 6), 28 (sham *n* = 4; SNx *n* = 6), and 84 days (sham *n* = 4; SNx *n* = 6) post-surgery. Biochemical measurements (creatinine clearance, proteinuria, and albuminuria) and histopathology were analyzed at these time points to evaluate kidney function and fibrosis.

#### Streptozotocin with uninephrectomy model of diabetic nephropathy

Male Wistar rats had a right UNx. Seven days post-surgery, STZ (35 mg/kg) was administered in sodium citrate buffer (0.1 M, pH 4.0) by intravenous injection into the tail vein using a 23G butterfly cannula (Venisystems, UK). After 2 days, glycemia was measured using a One Touch Basic glucose meter (LifeScan, Pennsylvania, USA). Animals with serum glucose concentrations > 20 mM were considered diabetic. Glycemia was controlled to 20–25 mM by titrating subcutaneous insulin implants (Linshin, Ontario, Canada) to prevent animal wasting. Sham-operated rats (1 month *n* = 4; 4 months *n* = 4; 8 months *n* = 4) that received 0.3 mL sodium citrate solution (0.1 M, pH 4.0) were used as controls. Creatinine clearance, albuminuria, glycemia, and histopathology were analyzed at 1 (*n* = 4), 4 (*n* = 8), and 8 (*n* = 8) months post STZ administration [43].

### Human chronic kidney disease cohort

Eligible patients and HV aged > 18 years were recruited at the Sheffield Kidney Institute (SKI CKD Biorepository, Sheffield, UK) and urine and blood samples were prospectively collected. Urine and serum samples were collected at routine outpatient clinics or as an inpatient for biopsy. No time restrictions were placed on urine collection. Urine was immediately placed on ice, then centrifuged at 10,000 x g for 15 minutes to remove cells/debris before being aliquoted and stored at -80°C within 2 hours of collection. Serum (non-fasting) was collected in a vacutainer, allowed to clot for 30 minutes before being cooled on ice, aliquoted and frozen at -80°C within 2 hours.

Key exclusion criteria were renal replacement therapy (dialysis or transplantation), acute kidney injury (AKI) at presentation, or urinary tract infection (UTI). CKD progression was evaluated as a function of the rate of eGFR decline calculated by the linear regression analysis of ≥ 10 eGFR values obtained over a period of approximately 3 years. Patients were stratified by eGFR decline (stable < 2 mL/min/year), progressive 2–5 mL/min/year and rapidly progressive (> 5 mL/min/year). HVs and patients provided written informed consent and the study protocol was approved by the South Humber Local Research

Ethics Committee under reference number 08/H1305/64 until October 2012 and 12/YH/0297 after this date. All work was performed under NHS R&D number STH16448.

### Biochemical measurements

Clinical chemistry was undertaken by the Clinical Chemistry Department at the Northern General Hospital (Sheffield, UK) using standard autoanalyzer methods. In rats, total protein excretion was measured by a modified Lowry protein assay [45], and albuminuria by a commercial enzyme-linked immunosorbent assay (ELISA) kit (Bethyl Laboratories, Texas, USA).

### Histopathology

Kidneys were harvested and fixed in 10% neutral buffered formalin solution (Sigma-Aldrich, Poole, UK) for 24 hours at 4°C before embedding in paraffin blocks. Sections were stained with Masson’s Trichrome (Department of Histopathology, Northern General Hospital, Sheffield, UK), 10 x 100x fields acquired on an Olympus BX 61 fluorescent microscope and multiphase image analysis performed using multiphase image AnalySIS™ 3.2 software (Soft Imaging Systems, Germany) to assess fibrosis levels.

### Transglutaminase 2 sandwich ELISA

Urinary and serum TG2 was measured by an in-house sandwich ELISA using a polyclonal goat anti-human TG2 (Abcam, Cambridge, UK) capture antibody and CUB7402 (Abcam) as a detection antibody. The same ELISA was used for human and rat samples of urine and serum. A TG2 standard curve ranged from 31.25 to 2000 pg/mL of human or rat TG2, dependent on samples being assayed. Rat urine samples were diluted 1:10, 1:50 and 1:100, and human urine samples diluted 1:1, 1:2 or 1:5. Serum samples (rat and human) were diluted 1:20 and 1:50. No pre-treatment of urine or serum occurred. Inter- and intra-assay coefficient of variation (CV) were 1.2% and 3.9% respectively using random blind replicate samples. The limit of detection was 31.25 pg/mL. Color development proceeded for 10 mins and the plate read on a Labsystems Multiskan Ascent using Genesis light software to curve fit (cubic spline equation).

### Quantitative measurement of ε-(γ-glutamyl)-lysine dipeptide

Protein (5 mg) was precipitated from urine with 10% (w/v) trichloroacetic acid. Ether extraction was performed to remove lipids. The protein was re-suspended in NH_4_CO_3_ buffer; a crystal of thymol was added to prevent bacterial growth before exhaustive proteolytic digestion (subtilisin, pronase E, leucine aminopeptidase, prolidase, and carboxypeptidase Y). The digest was freeze-dried, resuspended in 500 μL of lithium loading buffer (Biochrom, UK) and fractionated on a Biochrom 30 Amino Acid Analyzer. ε-(γ-glutamyl)-lysine produced a distinct peak at 570 nm with a retention time of around 77 mins. Quantification was by reference to a standard physiological amino acid mixture (Sigma) to which ε-(γ-glutamyl)-lysine standard (5 nmol/20 μL) was added.

### In vitro crosslinking of transglutaminase 2 substrates

Several proteins found in urine, including ECM proteins, were incubated with TG2 and the amount of ε-(γ-glutamyl)-lysine crosslinking was assessed. Recombinant human TG2 (10 μg) was added to 5 mM Tris buffer (pH 7.6) containing 2 mM CaCl_2_ and added to a 1 mg/mL solution (10 mM Ca^2^ Cl_2_, Tris pH 7.6, 0.1 mM dithiothreitol [DTT]) of the following proteins: human serum albumin, human collagen (types I, III, IV, and V), fibronectin from human plasma, and dimethyl casein (DMC). Following incubation with TG2 overnight at 37°C, each protein was subjected to exhaustive proteolytic digestion and the amount of ε-(γ-glutamyl)-lysine crosslink was quantified on a Biochrom 30 Amino Acid Analyzer as above. Human collagen I without the addition of TG2 was used as a negative control.

### Statistical analyses

One-way or two-way analysis of variance (ANOVA; as independent categorical groups) with Bonferroni correction (TG2 and ε-(γ-glutamyl)-lysine data were normally distributed, alpha 0.05) was applied to estimate differences between experimental groups as appropriate. A logistic regression model of CKD progression (any progression vs. stable disease) was built to assess the independent contribution of urinary TG2 and ε-(γ-glutamyl)-lysine in predicting progression. The model was adjusted for age, sex, CKD stage, UACR, and urine protein:creatinine ratio (UPCR). The effects of urinary TG2 and ε-(γ-glutamyl)-lysine were assessed after correcting for urine creatinine and transforming the data using a log_2_ transformation.

ROC curve analysis using DeLong’s empirical method was performed to compute area under the curve (AUC) for urinary TG2:creatinine ratio (UTCR) and urinary ε-(γ-glutamyl)-lysine:creatinine ratio (UXCR), and to determine the accuracy of predicting progression. Statistical analysis was performed using Microsoft Excel 2013 software package, GraphPad Prism 6 (GraphPad software Inc, California, USA) and R 3.6.1 (R project, https://www.r-project.org/). Statistical significance was considered if *P* < 0.05.

## Results

### Experimental renal scarring

In both SNx and DN models, progressive renal fibrosis occurred from 3 to 8 months respectively (S1 Fig in S1 File). Multiphase image analysis of Masson’s trichrome stained sections showed increasing glomerulosclerosis, which was associated with declining renal function and increasing proteinuria (S2 Fig in S1 File).

**Fig 1:**
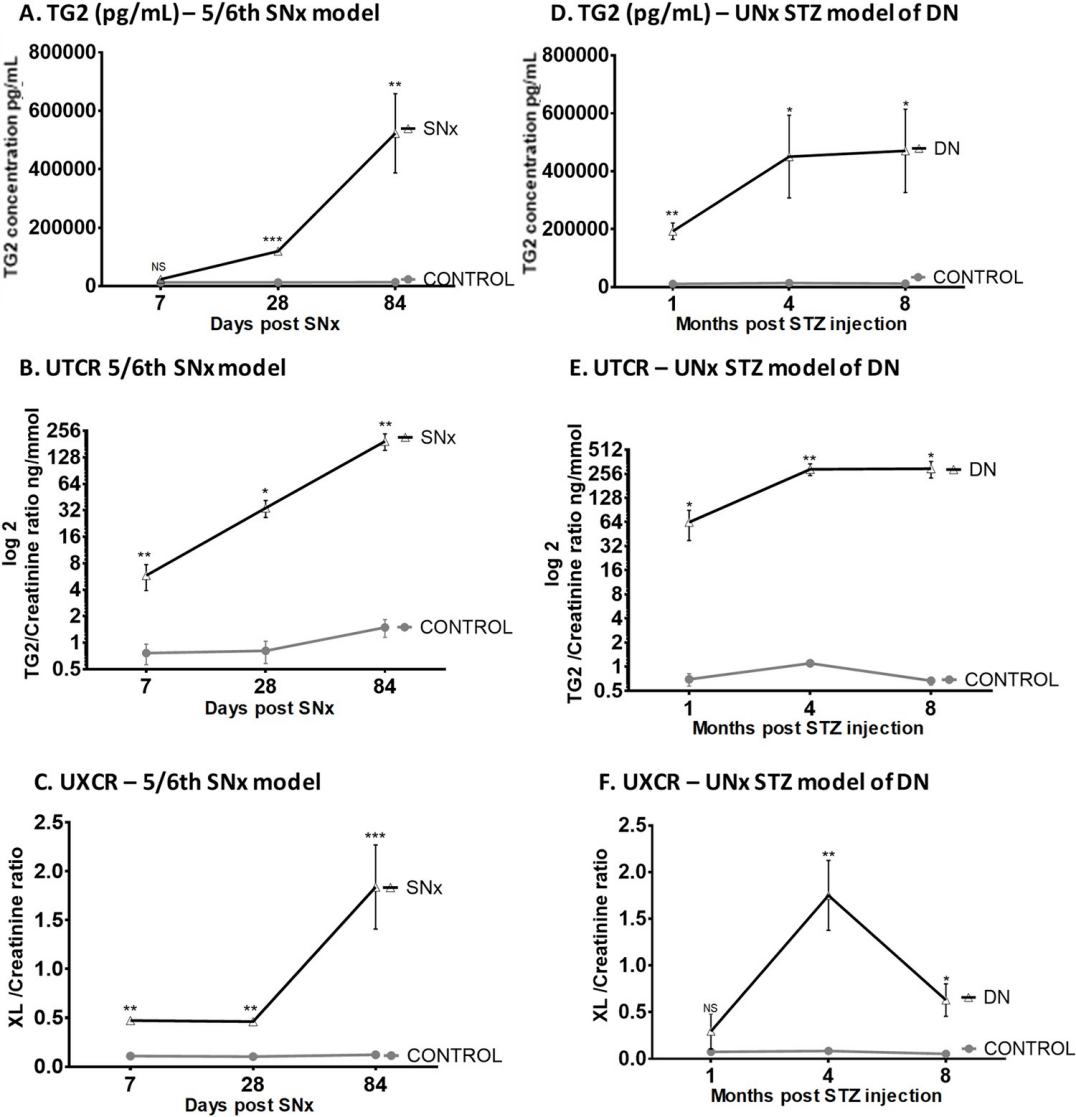
Urinary TG2 and ε-(γ-glutamyl)-lysine measurements in experimental kidney scarring. TG2 excretion was measured by ELISA and ε-(γ-glutamyl)-lysine crosslink (Glu-Lys) was measured in protein precipitates from rat urine samples by cation exchange chromatography. Urinary TG2 levels are shown by concentration (mean ± SEM, pg/mL) (A) and creatinine ratio (B) in the SNx model and repeated in the UNx STZ DN model, respectively (C, D). The UXCR was calculated in the UNx STZ (E) and DN (F) models. Statistical significance is shown by two-way ANOVA with Bonferroni post hoc test:, *P < 0.05, **P < 0.01, ***P < 0.001 between SNx or DN and normal urine. DN = diabetic nephropathy; ELISA = enzyme-linked immunosorbent assay; NS = not statistically significant; SEM = standard error of the mean; SNx = 5/6th sub-total nephrectomy; STZ = streptozotocin; TG2 = transglutaminase 2; UNx = uninephrectomy; UTCR = urinary TG2:creatinine ratio; UXCR = urinary ε-(γ-glutamyl)-lysine:creatinine ratio; XL = ε-(γ-glutamyl)-lysine crosslink.

In the SNx model, 24-hour albuminuria was significantly elevated at all time points (S2E Fig in S1 File). Urinary TG2 levels were elevated by 83% compared with control animals as early as Day 7 post-surgery, although this did not achieve statistical significance until Day 28, when levels were 9.3-fold higher than the time-matched controls (*P* = 0.0004). Urinary TG2 levels peaked at Day 84 and were 38-fold higher than control (*P* = 0.0045; Fig 1A). The UTCR was 7.6-fold higher in the SNx model compared with the control group by 1 week, 42-fold higher by 1 month, and 130-fold higher at 3 months post-surgery *(P* < 0.05, Fig 1B). Seven days post-SNx, the UXCR was significantly increased in the SNx group compared with controls (*P* = 0.0005; Fig 1C). This increase remained unchanged at 1 month with a similar 4.5-fold increase (*P* = 0.0002), before reaching a 15.2-fold increase at 3 months (*P* < 0.0001; Fig 1C).

In the DN model, 24-hour albuminuria was not significantly elevated until 8 months (S2F Fig in S1 File). In contrast, urinary TG2 concentrations were significantly higher than controls at all time points (Fig 1D). The UTCR steadily increased in DN animals throughout the time course showing 91-, 265-, and 443-fold increases at 1, 4, and 8 months, respectively (*P* < 0.05; Fig 1E). The UXCR was 4-, 21-, and 12-fold higher in DN animals at 1, 4, and 8 months, respectively, post-STZ injection compared with control animals (Fig 1F). In this model, the maximum UXCR was observed with an average of 1.75 ± 0.37 nmol/mg/mmol at 4 months.

### Human chronic kidney disease data

#### Patient demographics and disease characteristics

The cohort consisted of 290 patients with CKD and 33 HV; 88.2% were Caucasian and 62.5% males (Table 1; S3 Fig in S1 File). The median age of patients with CKD was 63.9 years (25^th^ percentile: 57 years, 75^th^ percentile: 76 years) and 61.9% were aged > 65 years. The patient distribution of CKD stages was: 3.8% stage 1, 6.9% stage 2, 38.6% stage 3, 45.5% stage 4, and 4.5% stage 5. Primary etiologies were: diabetic kidney disease (DKD, *n* = 90, 31.0%), chronic glomerulonephritis (CGN, *n* = 65, 22.4%), hypertensive nephrosclerosis (HTN, *n* = 53, 18.3%), atherosclerotic renovascular disease (ARVD, *n* = 29, 10.0%), chronic interstitial nephritis (CIN, *n* = 15, 5.2%), and autosomal dominant polycystic kidney disease (ADPKD, *n* = 13, 4.5%). Other causes of CKD (*n* = 25, 8.6%) included cases of cast nephropathy (multiple myeloma), amyloidosis, immune thrombocytopenia, Henoch Scholein purpura, monoclonal gammopathy of undetermined significance, microscopic polyarteritis, familial renal disease, medullary sponge kidney, sarcoidosis, lupus nephritis, HIV-associated nephritis, tacrolimus toxicity, and renal cell carcinoma (Table 1).

**Table 1:** Clinical and laboratory characteristics of healthy volunteers and patients with CKD.

Variable	HVs	Total CKD patients	DKD	CGN	HTN	ARVD	CIN	ADPKD	Others
**Number (*n*)**	33	290	90	65	53	29	15	13	25
**Gender:**	23 (69.7)	179 (61.7)	57 (63.3)	41 (63.1)	31 (51.5)	22 (75.9)	7 (46.7)	7 (53.9)	14 (56)
**Male, *n* (%)**									
**Age (years)**	38.5 ± 7.6	63.9 ± 16.6	68.2 ± 11.6	49.8 ± 17.8	71.6 ± 12.0	75.4 ± 6.4	62.1 ± 13.1	47.2 ± 17.9	61.9 ± 19.1
**Race:**	21 (63.6)	264 (91.0)	83 (92.2)	57 (87.7)	46 (86.8)	27 (93.1)	15 (100)	13 (100)	23 (92)
**Caucasians, *n* (%)**									
**BMI (kg/m** ^**2**^ **)**	22.3 ± 2.7	30.8 ± 6.0	31.8 ± 5.6	29.3 ± 5.7	31.2 ± 8.9	29.3 ± 4.0	27.9 ± 3.2	29.4 ± 6.6	25.9 ± 2.6
**Smokers, *n* (%)**	1 (3.0)	33 (11.3)	9 (10.0)	3 (4.6)	8 (15.1)	6 (20.7)	2 (13.3)	2 (15.4)	3 (12)
**CKD Stage 1, *n* (%)**	NA	11 (3.8)	0	9 (13.8)	0	1 (3.4)	0	1 (7.7)	0
**CKD Stage 2, *n* (%)**	NA	20 (6.9)	0	12 (18.5)	2 (3.8)	2 (6.9)	0	3 (23.1)	2 (8)
**CKD Stage 3A, *n* (%)**	NA	39 (13.4)	13 (14.4)	14 (21.5)	5 (9.4)	2 (6.9)	1 (6.7)	2 (15.4)	2 (8)
**CKD Stage 3B, *n* (%)**	NA	73 (25.2)	25 (27.8)	13 (20.0)	13 (24.5)	9 (31.0)	5 (33.3)	2 (15.4)	5 (20)
**CKD Stage 4, *n* (%)**	NA	132 (45.5)	49 (54.4)	15 (23.1)	28 (52.8)	12 (41.4)	8 (53.3)	4 (30.8)	15 (60)
**CKD Stage 5, *n* (%)**	NA	13 (4.5)	3 (3.3)	2 (3.1)	5 (9.4)	3 (10.3)	1 (6.7)	1 (7.7)	1 (4)
**eGFR at sampling (mL/min/1.73m** ^**2**^ **)**	>90	36.7 ± 21.2	30.9 ± 11.0	55.1 ± 29.9	29.5 ± 14.4	30.2 ± 18.1	28.4 ± 9.7	46.2 ± 31.0	37.1 ± 20.2
**CKD progression (%)**	NA	107 (36.9)	41 (45.6)	28 (43.1)	20 (37.7)	6 (20.7)	5 (33.3)	2 (15.4)	6 (22.6)
**Serum creatinine (μmol/L)**	81.1 ± 12.5	183.4 ± 74.7	188.2 ± 3.6	143.4 ± 6.9	208.2 ± 7.9	207.3 ± 5.7	188.8 ± 6.5	177.6 ± 2.7	185.8 ± 8.4
**Urine creatinine (μmol/L)**	11.4 ± 6.1	7.2 ± 4.9	6.5 ± 3.6	9.5 ± 6.6	7.8 ± 5.9	6.58 ± 4.1	6.0 ± 5.2	5.2 ± 4.2	6.8 ± 3.0
**Albuminuria (mg/L)**	1.8 ± 3.7	540.4 ± 1207.7	351.6 ± 633.2	1512.5 ± 2273.1	362.3 ± 611.8	296.8 ± 651.8	113.5 ± 218.6	165.4 ± 388.6	303.8 ± 505.9
**Proteinuria (mg/L)**	0.06 ± 0.03	0.9 ± 1.6	0.7 ± 1.0	2.0 ± 3.0	0.8 ± 0.9	0.5 ± 1.0	0.5 ± 0.6	0.4 ± 0.5	0.7 ± 1.1
**Serum cholesterol (mmol/L)**	NA	4.5 ± 1.6	3.8 ± 1.1	6.0 ± 2.3	4.6 ± 1.3	4.4 ±1.2	4.7 ± 1.4	4.3 ± 0.8	5.0 ± 1.2
**Triglycerides (mmol/L)**	NA	2.0 ± 1.4	1.9 ± 0.1	2.4 ± 1.8	2.1 ±1.9	1.8 ± 1.2	1.7 ± 1.0	1.6 ± 1.0	2.2 ± 1.3
**Serum calcium (mmol/L)**	NA	2.3 ± 0.1	2.3 ± 0.1	2.4 ± 0.1	2.3 ± 0.1	2.3 ± 0.1	2.3 ± 0.1	2.3 ± 0.1	2.3 ± 0.1
**Serum phosphorus (mmol/L)**	NA	1.2 ± 0.2	1.2 ± 0.3	1.2 ± 0.2	1.2 ± 0.2	1.3 ± 0.3	1.2 ± 0.2	1.3 ± 0.3	1.2 ± 0.2
**Ca x P (mmol** ^**2**^ **/L** ^**2**^ **)**	NA	2.8 ± 0.5	2.8 ± 0.5	2.9 ± 0.5	2.7 ± 0.5	3.0 ± 0.7	2.8 ± 0.5	2.9 ± 0.6	2.7 ± 0.5
**PTH (pmol/L)**	NA	146.6 ± 111.2	155.7 ± 112.0	93.1 ± 75.3	172.0 ± 131.0	148.8 ± 93.6	132.4 ± 94.0	149.8 ± 126.2	139.2 ± 109.9
**BUN (mmol/L)**	4.4 ± 1.2	13.9 ± 6.7	14.1 ± 6.7	12.7 ± 4.6	13.8 ± 4.9	13.2 ± 5.1	12.1 ± 4.9	13.2 ± 5.8	13.9 ± 6.9

Data are given as total number (n), percentage of total study population (%) and as mean ± SD, where applicable. Other causes of CKD included cases of: cast nephropathy (multiple myeloma), amyloidosis, idiopathic thrombocytopenic purpura, Henoch Scholein purpura, monoclonal gammopathy of undetermined significance (MGUS), microscopic polyarteritis, familial renal disease, medullary sponge kidney, sarcoidosis, lupus nephritis, HIV-associated nephritis, tacrolimus toxicity and renal cell carcinoma.

ADPKD, adult polycystic kidney disease; ARVD, atherosclerotic renovascular disease; BMI, body mass index; BUN, blood urea nitrogen; Ca, calcium; CGN, chronic glomerulonephritis; CKD, chronic kidney disease; CIN, chronic interstitial nephritis; DKD, diabetic kidney disease; eGFR, estimated glomerular filtration rate; HTN, hypertensive nephrosclerosis; HV, healthy volunteers; NA, not applicable; P, phosphorus; PTH, parathyroid hormone; SD, standard deviation.

Over the duration of the study, the mean rate of eGFR decline for the entire CKD population was –0.79 mL/min/1.73m^2^. Around one-third of patients were classified as progressive (eGFR decline > –2 mL/min/1.73m^2^/year) and 184 patients were classified as stable or non-progressive (eGFR decline < –2 mL/min/1.73m^2^/year). Of the progressive patients, 26.9% had a more rapid progression (> 5 mL/min/1.73m^2^/year).

#### Urinary transglutaminase 2 concentration

**Fig 2:**
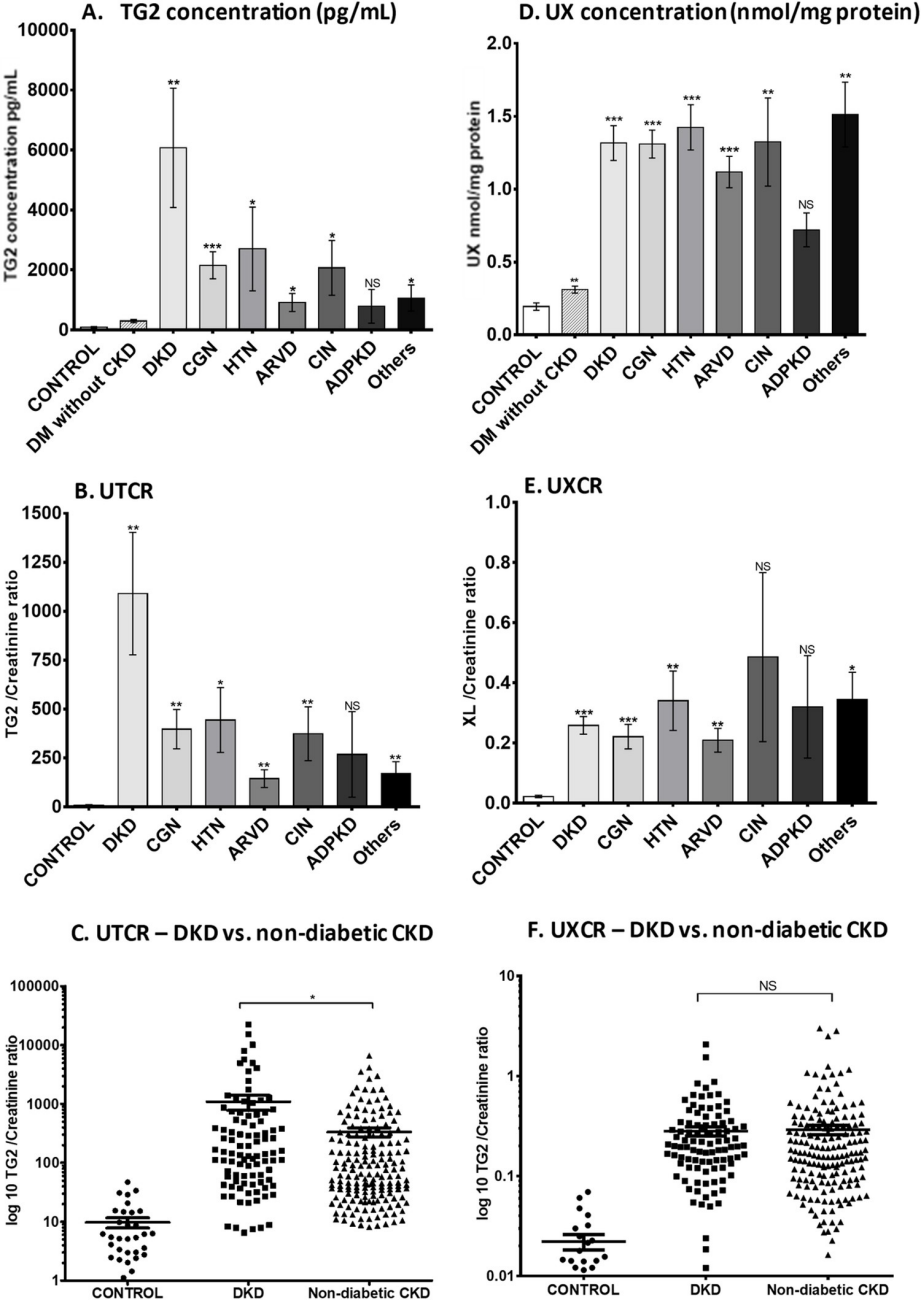
Urinary TG2 and ε-(γ-glutamyl)-lysine excretion in patients with different causes of CKD. Urinary TG2 was measured by an in-house ELISA in patients with CKD (total *n* = 290), healthy volunteers (*n* = 33) and expressed as a concentration (ng/mL) (A), then corrected to a creatinine ratio (D) with variation displayed as a scatter plot of UTCR in DKD (*n* = 90) and non-diabetic CKD patients (*n* = 200), with y axis in log 10 scale (B). ε-(γ-glutamyl)-lysine crosslink (Glu-Lys) was measured in protein precipitates from human urine samples by cation exchange chromatography and displayed as mean Glu-Lys per mg urine protein ± SD calculated by AUC of peaks (E) and then corrected to a creatinine ratio (C) using a log 10 scale. Variation is shown using a scatter plot of UXCR in DKD and non-DKD CKD. Results are displayed as mean ± SD. Statistical significance is shown by one-way ANOVA with Bonferroni post hoc test: *P < 0.05, **P < 0.01, ***P < 0.001 between CKD patients and HV. ADPKD = autosomal dominant polycystic kidney disease; ARVD = atherosclerotic renovascular disease; AUC = area under the curve; CGN = chronic glomerulonephritis; CIN = chronic interstitial nephritis; CKD = chronic kidney disease; DKD = diabetic kidney disease; DM = diabetes mellitus; ELISA = enzyme-linked immunosorbent assay; HTN = hypertensive nephrosclerosis; HV = healthy volunteer; NS = not statistically significant; SD = standard deviation; TG2 = transglutaminase 2; UTCR = urinary TG2:creatinine ratio; UX = urinary ε-(γ-glutamyl)-lysine; UXCR = urinary ε-(γ-glutamyl)-lysine:creatinine ratio.

TG2 levels in urine were 35 times higher in the whole CKD patient population (3346 ± 758 pg/mL) than in HVs (94 ± 19 pg/mL, *P* < 0.0001). Urinary TG2 concentrations were higher in patients with diabetes without kidney disease (295 ± 53 pg/mL) compared with HVs (*P* = 0.0016; Fig 2A), but were significantly lower than the CKD patients (*P* < 0.0001); those with DKD (*P*< 0.01) averaged the highest TG2 concentration (6071 ± 1989 pg/mL, *P* = 0.0034).

**Fig 3:**
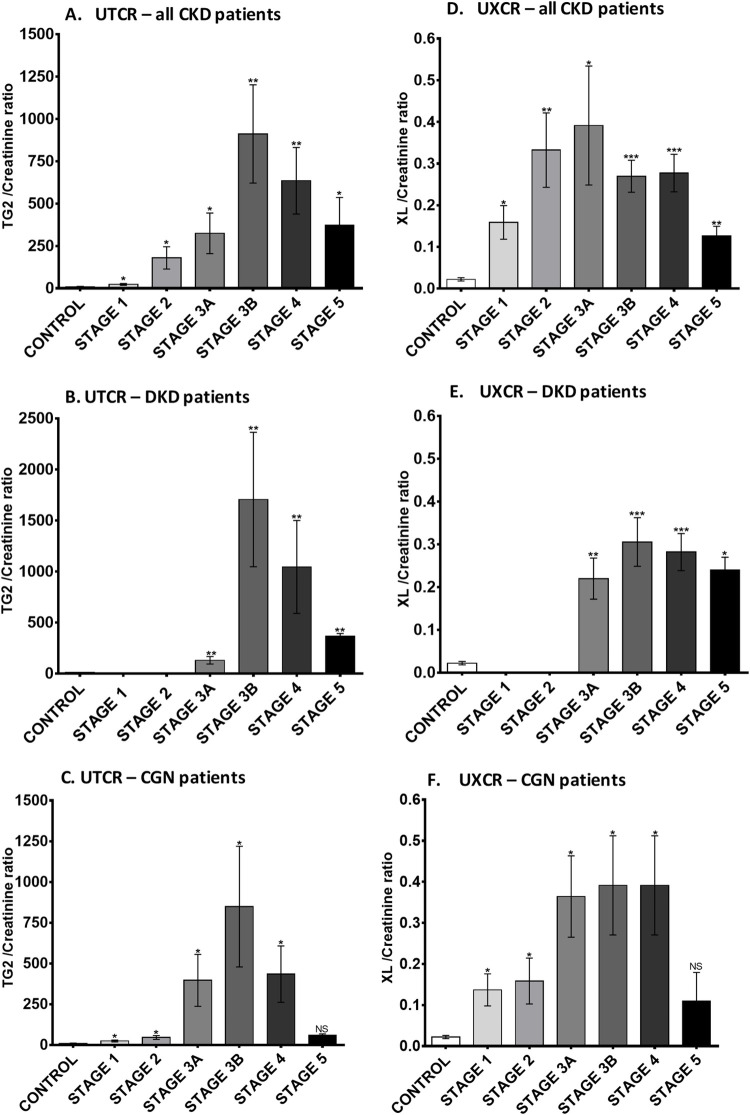
Urinary TG2 and ε-(γ-glutamyl)-lysine excretion in CKD patients at different stages. Average UTCR (A) and UXCR (D) in all CKD patients subdivided by the disease stages. Control group, *n* = 33; CKD stage 1, *n* = 11; CKD stage 2, *n* = 20; CKD stage 3A, *n* = 39; CKD stage 3B, *n* = 73; CKD stage 4, *n* = 132; CKD stage 5, *n* = 13. (B) and (E) show data from DKD patients, (C) and (F) show data from CGN patients. Statistical significance is shown by one-way ANOVA with Bonferroni post hoc test: *P < 0.05, **P < 0.01, ***P < 0.001 between CKD patients and HV. Data are mean ± SD. CGN = chronic glomerulonephritis; CKD = chronic kidney disease; DKD = diabetic kidney disease; HV = healthy volunteer; NS = not statistically significant; SD = standard deviation; TG2 = transglutaminase 2; UTCR = urinary TG2:creatinine ratio; UXCR = urinary ε-(γ-glutamyl)-lysine:creatinine ratio; XL = ε-(γ-glutamyl)-lysine crosslink.

The UTCR was elevated in all types of CKD compared with HVs (Fig 2B), apart from ADPKD which showed large patient variability. However, there were differences between etiologies; the greatest increase was in DKD (80-fold), which was double that of other CKD types, including CGN (40-fold), HTN (45-fold), ARVD (14-fold), CIN (38-fold), and other causes of CKD (17-fold). Scatter plots demonstrated minimal overlap of UTCR values between HV and those with both diabetic and non-diabetic CKD (Fig 2C). Breakdown of UTCR by stage showed peak levels at stage 3B, which reduced to roughly one-third of that by stage 5 (Fig 3A). This was consistent irrespective of etiology (exemplified by DKD [Fig 3B] and CGN [Fig 3C]).

**Fig 4:**
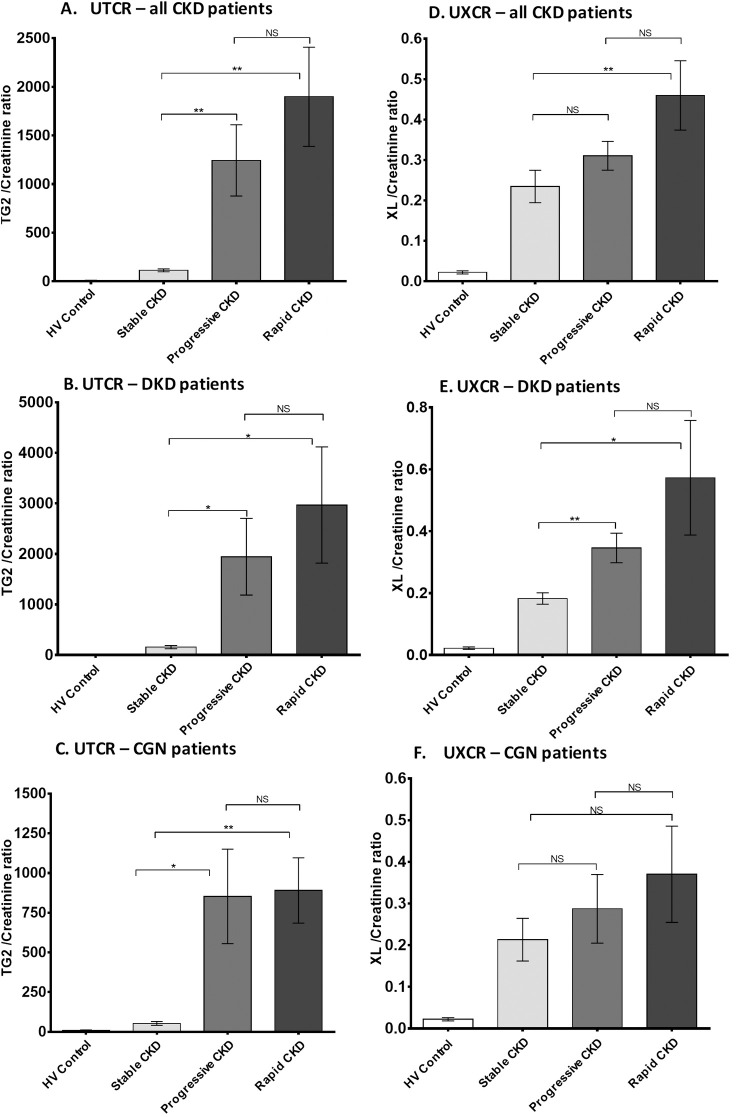
Urinary TG2 and ε-(γ-glutamyl)-lysine excretion in CKD patients by disease progression. UTCR and UXCR measurements in CKD patients were subdivided into groups according to their rate of disease progression: non-progressive (rate of eGFR decline < 2 mL/min/1.73m^2^/year, *n* = 184), progressive (between 2–5 mL/min/1.73m^2^/year, *n* = 79), and rapidly progressive (> 5 mL/min/1.73m^2^/year, *n* = 29) and data presented as a bar chart in all CKD patients (A) and (D), DKD patients (B) and (E), and CGN patients (C) and (F). Statistical significance is shown by one-way ANOVA with Bonferroni post hoc test: *P < 0.05, **P < 0.01, ***P < 0.001 between non-progressive, progressive, and rapidly progressive patients. Data are mean ± SD. CGN = chronic glomerulonephritis; CKD = chronic kidney disease; DKD = diabetic kidney disease; eGFR = estimated glomerular filtrate rate; HV = healthy volunteer; NS = not statistically significant; SD = standard deviation; UTCR = urinary TG2:creatinine ratio; UXCR = urinary ε-(γ-glutamyl)-lysine:creatinine ratio; XL = ε-(γ-glutamyl)-lysine crosslink.

Compared with patients with stable CKD (*n* = 183), a significantly higher UTCR was reported in patients with progressive CKD (*n* = 78, *P* = 0.0029) and rapidly progressive CKD (*n* = 29, *P* = 0.0017; Fig 4A). This increase in UTCR in progressive patients was consistent across all types of CKD (exemplified by DKD [Fig 4B] and CGN [Fig 4C]).

#### Urinary ε-(γ-glutamyl)-lysine concentration

Urinary ε-(γ-glutamyl)-lysine was approximately 6-times higher in all types of CKD compared with HVs (Fig 2D). The UXCR ratio was 12.5-times higher in patients with all-cause CKD (0.2796 ± 0.0285 nmol/mg/mmol) than HVs (0.0222 ± 0.004 nmol/mg/mmol, *P* < 0.001); there was little variability between CKD types (Fig 2E) but clear separation from HVs with < 10% of CKD patients in the normal range (Fig 2F). The UXCR in patients at different CKD stages was significantly increased in the whole CKD population (*P*<0.05; Fig 3D). The UXCR peaked at stage 3A, with levels progressively declining by more than 66% at stage 5. There were some differences in level and stage of detection between different diseases, exemplified by DKD (Fig 3E) and CGN (Fig 3F).

The UXCR was 12-fold higher in non-progressive CKD patients compared with HVs (Fig 4D). There was a small but notable increase in those with progressive disease (15-fold). Patients with rapid progression had a mean approximately 30-fold above normal; however, the large range meant that this was not significantly elevated over the progressive patient cohort and only significant compared to patients with stable disease (*P* = 0.0262). This increase in UXCR in progressive patients was not consistent when broken down by etiology (e.g., significant changes can be seen in DKD, but not CGN [Fig 4E and 4F]).

#### Serum transglutaminase 2 concentration

TG2 was also measured in serum samples from a subset of 130 patients with CKD (DKD *n* = 58, CGN *n* = 24, HTN *n* = 23, ARVD *n* = 8, CIN *n* = 5, ADPKD *n* = 4, other *n* = 8) and 10 HVs in order to compare levels in the circulation with urine excretion.

**Fig 5:**
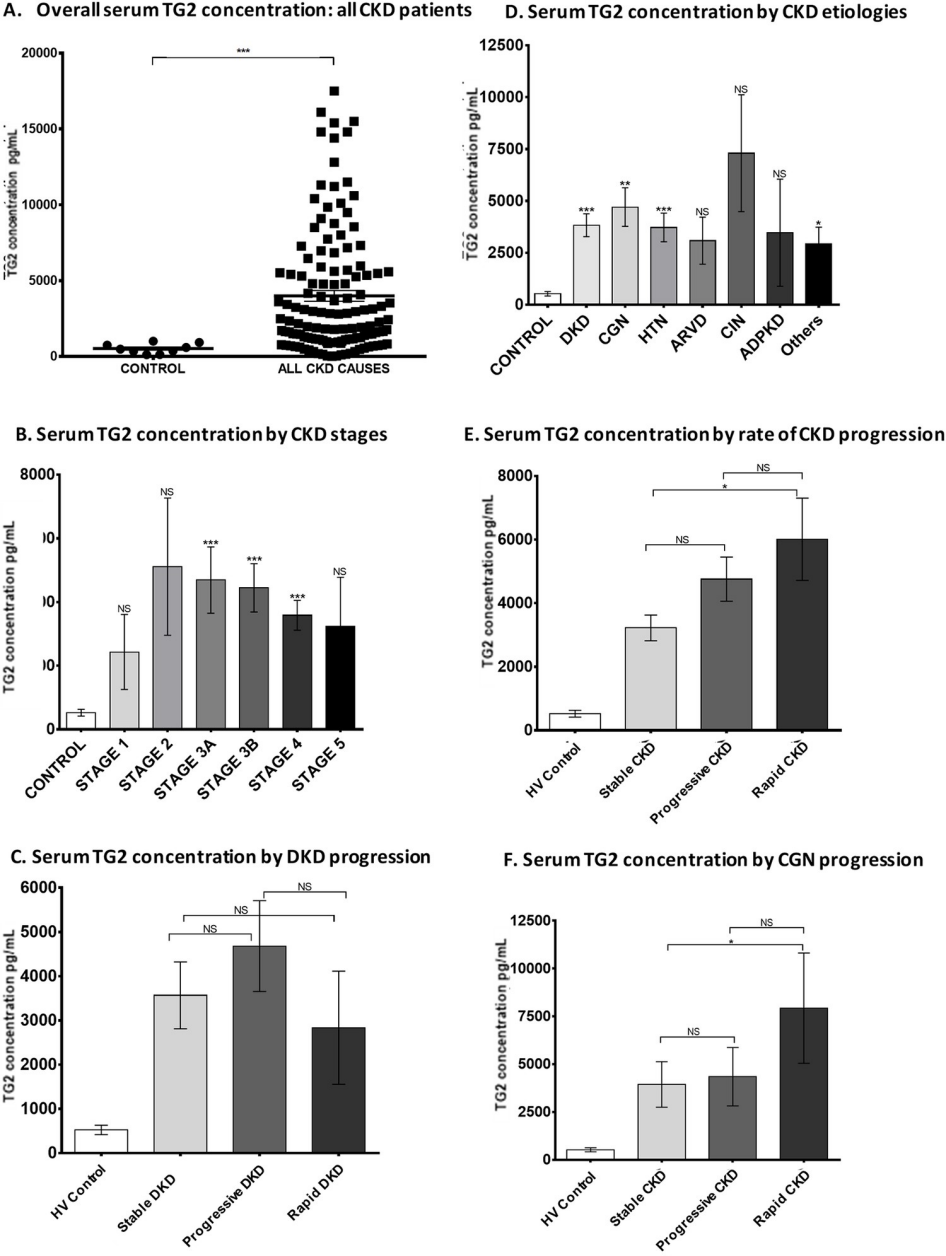
Serum TG2 concentration in patients with CKD. Serum TG2 concentration was quantified by an in-house sandwich ELISA. (A) Scatter plot of serum TG2 measurements in HV (*n* = 9) and CKD patients (*n* = 130), (D) average serum TG2 concentration (pg/mL) in different types of CKD, (B) at different CKD stages, (E) by rate of CKD progression, and in the two largest groups of patients present in the cohort: (C) DKD and (F) CGN. Statistical significance was shown by one-way ANOVA with Bonferroni post-hoc test: *P < 0.05, **P < 0.01, ***P < 0.001. Data are mean ± SD. CGN = chronic glomerulonephritis; CKD = chronic kidney disease; DKD = diabetic kidney disease; ELISA = enzyme-linked immunosorbent assay; HV = healthy volunteer; NS = not statistically significant; SD = standard deviation; TG2 = transglutaminase 2.

Average serum concentration of TG2 in HVs was 523 ± 107 pg/mL, which represented approximately 5-times the urine concentration (*n* = 33). Patients with CKD had a significantly higher serum concentration of TG2 compared with HVs (Fig 5A), with little difference between diseases (Fig 5D). While patients with DKD, CGN, HTN, and other CKD causes had significantly higher serum TG2 levels than HVs, no significant difference was found in patients with ARVD, CIN, or ADPKD, due to large variability within these groups (Fig 5D). Serum TG2 was less affected by stage than urinary TG2 (Fig 5B). Only patients with rapidly progressive disease had significantly higher serum TG2 levels than stable patients (Fig 5E) and this was not consistent across different types of CKD (exemplified by DKD [Fig 5C] and CGN [Fig 5F]).

#### Potential transglutaminase 2 substrates

To establish which urinary proteins could be substrates for TG2 crosslinking, proteins were incubated with TG2 and the amount of ε-(γ-glutamyl)-lysine crosslinking assessed (S4 Fig in S1 File). ε-(γ-glutamyl)-lysine was hardly detectable in human albumin (0.01 nmol/mg protein). Collagen III, DMC, fibronectin, and collagen I all had high capacity to be crosslinked by TG2.

#### Logistic regression and receiver operating characteristic curve analysis

**Fig 6:**
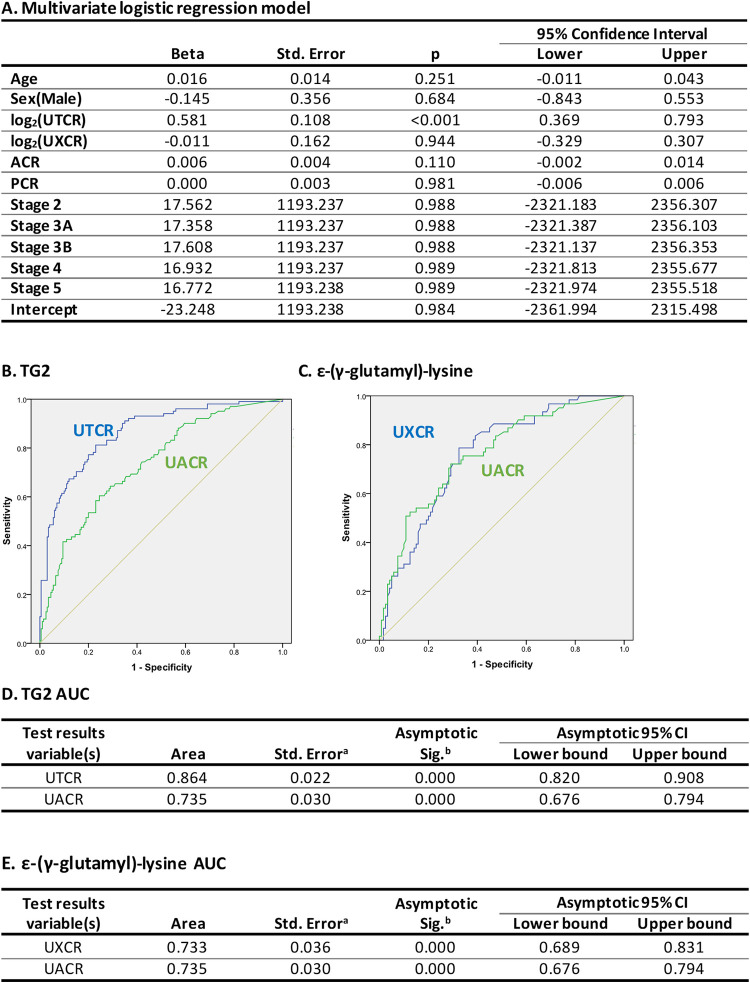
Multivariate logistic regression modelling and ROC curve analysis to evaluate the value of urinary TG2 and ε-(γ-glutamyl)-lysine to predict progression. (A) Multivariate logistic regression model for CKD progression vs. stable disease adjusted for UTCR and UXTR (for the purpose of estimation, the model was fit on the log_2_-transformed value of these variables). UTCR (B) and UXCR (C) ROC curves with sensitivity (probability of correctly identifying a positive finding) plotted on the y axis and 1-specificity (false positives) on the x axis. The higher the AUC (D, E for UTCR and UXTR respectively), the higher the accuracy of prediction of an event. ^a^Under the nonparametric assumption. ^b^Null hypothesis: true area = 0.5. ACR = albumin creatinine ratio; AUC = area under the curve; CKD = chronic kidney disease; PCR = protein creatinine ratio; ROC = receiver operating characteristic; Sig. = significance; Std. = standard; TG2 = transglutaminase 2; UACR = urinary albumin:creatinine ratio; UTCR = urinary TG2:creatinine ratio; UXCR = urinary ε-(γ-glutamyl)-lysine:creatinine ratio.

Both urinary TG2 and ε-(γ-glutamyl)-lysine were significantly associated with speed of progression in univariate logistic regression models (log_2_(UTCR), beta = 0.597, 95% confidence interval (CI) 0.442–0.752; *P* < 0.001; log_2_(UXCR), beta = 0.573, 95% CI 0.340–0.806; *P* < 0.001). In the multivariate model adjusted for urinary TG2, ε-(γ-glutamyl)-lysine, age, sex, UACR, UPCR, and CKD stage, only TG2 remained statistically significant (Fig 6A); thus, suggesting that the univariate signals of TG2 and ε-(γ-glutamyl)-lysine are the same. UACR and UPCR showed no significant effect in the multivariate regression model (Fig 6A) despite significant effects in the univariate analysis: UACR, beta = 0.005, 95% CI 0.003–0.007; *P* < 0.001; UPCR, beta = 0.004, 95% CI 0.002–0.006; *P* < 0.001. Correlation analysis also showed a lack of correlation between urinary TG2 and proteinuria (S5 Fig in S1 File).

ROC curve analysis showed that the UTCR from a single spot urine was 86.4% accurate in identifying those most likely to experience CKD progression over the following 3 years, compared with 73.5% based on UACR (Fig 6B and 6D). UXCR, however, did not display the same predictive power, being comparable with UACR (Fig 6C and 6E), and a combination of UTCR and UXCR showed no superiority. Additionally, using the ROC curve, the best cut-off value for UTCR to predict progression was determined as 25.576 by obtaining the value that maximized both the sum of the sensitivity and specificity (corresponding to the point closest to the upper left corner in the ROC curve; S6 Fig in S1 File). Serum TG2 concentrations were 64.7% accurate in predicting progression, compared with 68.0% for UACR (S7 Fig in S1 File).

## Discussion

In this study we measured urinary TG2 and its crosslink product ε-(γ-glutamyl)-lysine in biofluids from rat models and patients with CKD. Animal data suggested urinary TG2 had value as a predictive biomarker of renal fibrosis and decline, with levels significantly increased early in both SNx and DN models, showing better correlation to disease progression than proteinuria. Urinary TG2 changes mirrored those in tissue, consistent with previously reported observations in these models [33, 46]. In the SNx model, ε-(γ-glutamyl)-lysine also looked to be a promising biomarker, but in experimental DN, despite early increases, no changes were detected in samples at the 8-month timepoint, contrasting with our published tissue measures of ε-(γ-glutamyl)-lysine [46]. While this may suggest ε-(γ-glutamyl)-lysine changes seen in the kidney may not reach the urine, this was not the case for the SNx model or human samples. This may be due, in part, to the large volumes of dilute urine produced by diabetic animals, or the fact that urinary proteins present in late-stage DN animals are predominantly of plasma rather than renal origin. Consistent with that perspective, our results show albumin to be a poor TG2 substrate compared with ECM proteins and, as albumin forms a disproportionately large proportion of the urinary proteome, this would comparatively reduce ε-(γ-glutamyl)-lysine levels when measured as a fraction of total protein.

Urinary TG2 was elevated in both SNx and DN models at the first sampling points post disease induction, i.e. 7 and 28 days, respectively. In the SNx model, this offered little predictive advantage over albuminuria; however, in the DN model, changes in albuminuria were not notable at 1 and 4 months, respectively (first and second sampling points), and only substantially elevated at the final time points in these animals, and in other studies using this model [33, 46]. This suggests that TG2 could be altered early in the disease process and thus urinary TG2 may be an earlier marker of CKD. Consistent with this notion, we also observed significant changes in both urinary TG2 and ε-(γ-glutamyl)-lysine in CKD stage 1 and 2 patients with minimal proteinuria.

Urinary TG2 and ε-(γ-glutamyl)-lysine levels were elevated in CKD patient samples and were consistent with the pre-clinical model observations. Only patients with ADPKD, who presented without higher progressive renal function decline, were without significantly elevated urinary TG2. The UTCR was clearly elevated in progressive and rapidly progressive CKD compared with stable disease; however, this was not paralleled by ε-(γ-glutamyl)-lysine excretion, where only the rapidly progressive patients displayed significantly higher urinary levels of ε-(γ-glutamyl)-lysine than stable patients.

The multivariate logistic regression model showed that urinary TG2 is a strong predictor of progression (any progression vs. stable disease) and this effect is independent of albumin creatinine ratio (ACR), protein creatinine ratio (PCR), and CKD stage. Also, ACR and PCR are not significantly associated with speed of progression, thus suggesting that urinary TG2 is a better predictor than these two commonly used markers. In addition, ε-(γ-glutamyl)-lysine had no significant effect in the multivariate model, which is in agreement with the univariate model data.

ROC curve analysis was used to evaluate the predictive potential of urinary TG2 and ε-(γ-glutamyl)-lysine to identify patients at greater risk of progression. UTCR gave 13% greater accuracy than UACR in identifying progressive patients from a single spot urine. The UXCR, however, did not display the same power of prediction, similar to UACR. This suggests that the UTCR could be a better prognostic tool than the currently used methods; however, confirmation in multiple cohorts at different centers is required. Importantly, there was a lack of correlation between urinary TG2 and proteinuria, implying that increased urinary TG2 excretion was not simply about raised proteinuria *per se* generating higher urine levels. In our analysis, we determined the best cut-off value for UTCR that separated progressors from stable patients. Using UTCR alone and this cut-off, a diagnostic test to determine progression status (stable vs. progressor) would have a classification accuracy of 75.2%, a sensitivity of 79.8%, and a specificity of 72.3%.

This study also evaluated serum TG2 levels. If the same predictive potential as seen in urine could be achieved in blood, this would negate the need to correct for urine volume by creatinine. Surprisingly, serum levels of TG2 were 5-times higher than those found in urine, however, in contrast to urinary concentrations, serum concentrations were only able to distinguish between rapidly progressing and stable disease patients, not progressive and stable renal function.

These data suggest TG2, particularly in urine, may be a valuable tool in both early detection of CKD and importantly, identifying which patients may progress more rapidly. However, it also raises questions of how TG2 finds its way into serum and perhaps more pertinently, urine. We know from human [39, 47] and animal studies [33, 44, 48] that TG2 is hugely elevated in CKD, predominantly in the tubular epithelium—likely as part of its role as an early wound response enzyme to stabilize the ECM and activate TGFβ1 to drive cell proliferation. This potentially explains why TG2 may be an earlier marker of CKD damage, as elevated concentrations would occur ahead of protein leakage or functional decline which require significant remodeling to occur. A key part of this is a novel extracellular cell trafficking mechanism [49] that releases large amounts of stored TG2 into the extracellular space in response to stress [50]. It is therefore possible that the TG2 from the tubular epithelium is either externalized apically directly into the lumen, or more likely, basolaterally into the extracellular space around the tubular basement membrane [49]. Once in this area, leakage into the lumen in a compromised tubular structure is the most feasible route to urine. However, it is clear from these data that there is a higher concentration of TG2 in serum of patients with CKD, suggesting uptake from the extracellular space around damaged tubules. Given TG2 is approximately 75 to 85 kDa, while it would be unlikely to be filtered significantly in normal kidney, it is highly likely that in patients with proteinuria some filtration could occur. That said, there was no correlation of urine TG2 to proteinuria, and there are no published data on how well TG2 is reabsorbed from urine in health or disease. Fractional excretion studies would be highly valuable to improve our understanding and would need timed urine collection studies that are beyond the scope of the present study.

While the data presented here indicate the potential value of urinary TG2 as a prognostic or stratification biomarker in CKD, the study has limitations. While the TG2 ELISA had a CV of < 20%, acceptable linearity on dilution, and recovery from spiking experiments (92–113%), the assay is considered “research grade” due to some matrix effect. A larger multi-center cohort (to confirm these observations) and an improved assay will help to negate the major reasons for the failure of numerous novel CKD prognostic markers to translate to the clinic. It is also essential to understand if there are any factors/activities that affect TG2 values independently of renal disease, in a similar way physical activity can affect albuminuria. Currently we are not aware of any such confounders, but it is theoretically possible that fibrosis in other organs could have an impact.

Although the ε-(γ-glutamyl)-lysine data suggest that this marker is less attractive as a prognostic biomarker, it does show notably elevated levels in CKD and may offer potential as a pharmacodynamic/target engagement marker for future trials of TG2 inhibitors in fibrotic diseases; equally, the ε-(γ-glutamyl)-lysine assay used here requires further validation. The potential for variable digestion, the lower level of detection, and relatively small peak height from amino acid analysis are all factors that create significant variability. Digestion with immobilized proteolytic enzymes and a mass spectrometry endpoint are a clear development.

A comparison of urinary TG2 with other reported biomarkers of CKD progression is also desirable. Many other markers have been reported including kidney injury markers, such as NGAL [51, 52] and KIM-1 [15, 53], functional markers such as cystatin C [54], and inflammatory markers including CCL2 [55], TWEAK [22], TNFα [56], IL-6 [57], and soluble TNFR1/2 (sTNFR1/2) [58]. Low urinary epidermal growth factor has consistently been associated with poor outcomes [59] and is often measured in combination with CCL2. The molecular chaperone protein clusterin (apolipoprotein J) has also been linked to CKD progression, being higher in both diabetic and non-diabetic CKD [60]. In particular, type 2 DKD patients who have sTNFR1/2 values in the highest quartile were 84% and 78%, respectively, likely to reach end-stage disease within 12 years [61]. Extrapolation of these sTNFR data has led to development of the KRIS, consisting of 17 inflammatory proteins as a prognostic tool, and it will be important to determine how urinary TG2 performs alongside this panel. Studies around fibrosis-related markers such as TG2 have been performed, including urine procollagen III [62], and suggested an association with disease stage rather than progression. Matrix metalloproteinase activity [37] and antigen [63] suggest that levels of ECM proteases in both urine and blood could be prognostic. This is also seen with serine proteases such as soluble urokinase plasminogen activator receptor [64], especially in glomerular diseases. The NURTuRE consortium [65] will examine approximately 40 putative biomarkers reported in the literature in > 3800 patients, including TG2, allowing the first real evaluation of combination and relative individual value.

In addition to these targeted and hypothesis-driven biomarker studies, there are a number of unbiased proteomic studies being conducted in patients to identify a range of proteins as biomarkers of CKD, especially those that may be involved in matrix remodeling such as collagens [66]. Some have generated compelling associations with CKD progression rates [67], although full analysis of hits by classical assays is often lacking. Some proteomic approaches utilize large parts of the proteome to predict GFR loss, exemplified by the CKD273 MS panel [68]. There are also proteomic studies in animal CKD models that yield more focused data by concentrating on specific aspects of pathology that cannot be interrogated in patients [69], but can be subsequently associated with the natural history of disease in patients. Advances are being made to determine, as well as validate, sensitive and specific biomarkers of renal function decline as exemplified by the kidney risk inflammatory signature (KRIS [23]) and some of the Nordic Biosciences neoepitopes [70, 71], and it will be important to incorporate a measure of urinary TG2 alongside these evaluations.

In conclusion, we quantified the urinary levels of TG2 and ε-(γ‐glutamyl)-lysine in human CKD caused by different etiologies. TG2 and ε-(γ-glutamyl)-lysine are significantly elevated in urine from CKD patients independently of increased proteinuria. ROC curve analysis determined a superior prediction of CKD progression for UTCR (86.4%), to the best conventional marker (UACR, 73.5%). A urinary biomarker panel including TG2 and ε-(γ-glutamyl)-lysine could represent a more effective way of predicting progressive CKD, which would help clinical practice, and be beneficial for trial stratification, as well as determining relationship to clinical outcome. These data warrant further investigation in a larger multi-center cohort.

## Supporting information

S1 File (DOCX)

S1 Dataset (XLSX)
