# Supplementary material for: Urinary levels of pro-fibrotic transglutaminase 2 (TG2) may help predict progression of chronic kidney disease
Source: PLoS One. 2022 Jan 18;17(1):e0262104. doi: 10.1371/journal.pone.0262104 (PMC8765645; doi:10.1371/journal.pone.0262104)
Supplement: S1 File — (DOCX) [file pone.0262104.s001.docx]

**Supporting Figures**

**Urinary levels of pro-fibrotic transglutaminase 2 (TG2) may help predict progression of chronic kidney disease**

Michelle Da Silva Lodge^1^, Nick Pullen^2^, Miguel Pereira^3^ and Timothy S. Johnson^1^

^1^ Academic Nephrology Unit and Sheffield Kidney Institute, University of Sheffield Medical School, Sheffield, UK

^2^ Pfizer Global Research and Development, Cambridge, MA, USA

^3^ Statistical Sciences and Innovation, UCB Pharma, Slough, UK

**S1 Fig.** **Representative Masson’s Trichrome stained sections (200x magnification) of the 5/6^th^ sub-total nephrectomy (SNx: A-D) and diabetic nephropathy (DN: E-H) models.**

**A. Normal**

**B. 1 Week SNx**


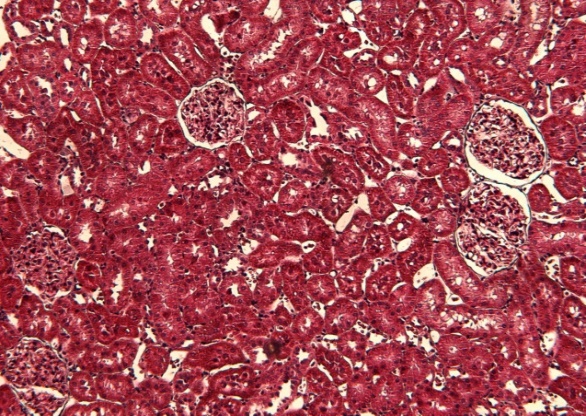

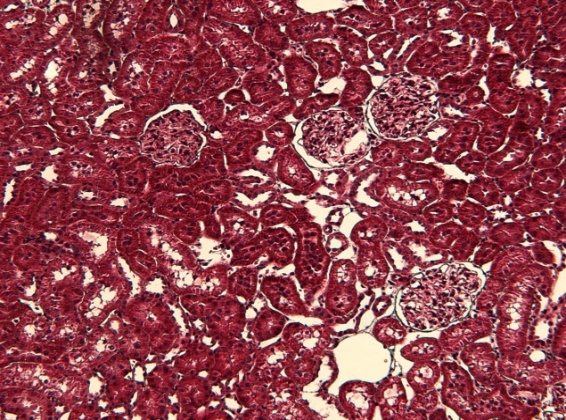


**C. 1 Month SNx**

**D. 3 Months SNx**


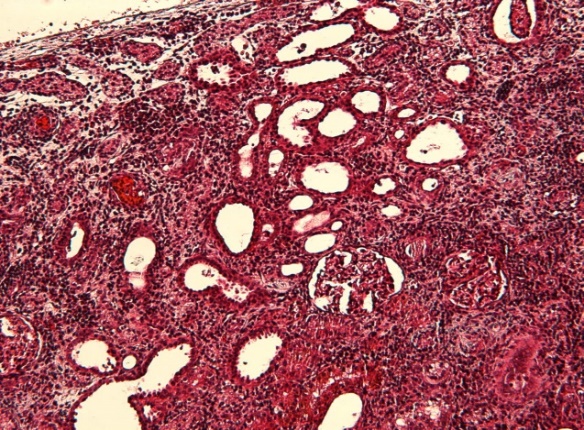

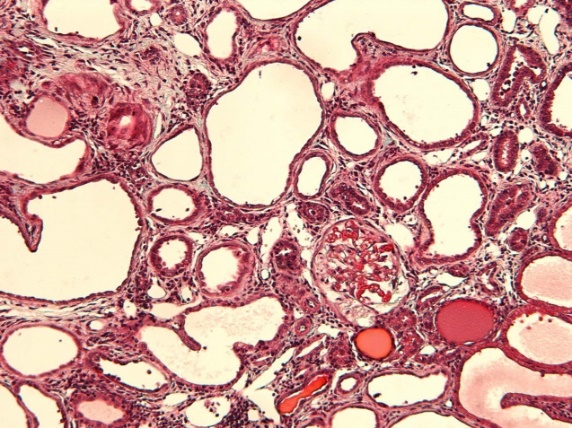


**F. 1 Month DN**

**E. Normal**

**H. 8 Months DN**

**G. 4 Months DN**

**S2 Fig.** **Development of disease in animal models of CKD.** The level of glomerulosclerosis (A B), renal function (C, D) and proteinuria (E, F) was assessed in SNx (A, C, E) and DN (B, D, F) models of CKD. The degree of glomerulosclerosis was assessed in Masson’s Trichrome stained sections by high content image analysis and a fibrotic index calculated**.** Function was measured by either creatinine clearance (SNx) or serum creatinine (DN). Albuminuria was measured per 24 hours. Data are mean ± SEM. **P* < 0.05, ***P* < 0.01, SNx or DN to sham group.
CKD, chronic kidney disease; DN, diabetic nephropathy; SEM, standard error of the mean; SNc, sham-operated control; SNx, 5/6^th^ sub-total nephrectomy.

**A. Glomerulosclerosis SNx** **B. Glomerulosclerosis DN**

7 28 84

**Days post SNx**

1 4 8

**Months post STZ**

1 4 8

Months post STZ

**C. Creatinine clearance SNx** **D. Serum creatinine DN**

1 4 8

**Months post STZ**

7 28 84

**Days post SNx**

**E. Albuminuria SNx** **F. Albuminuria DN**

7 28 84

**Days post SNx**

1 4 8

**Months post STZ**

**S3 Fig. Flow diagram of patient recruitment for the study**

**Followed for 3 years**

318 patients

**Urine baseline analysis 290 patients**

**Recruited**

347 patients

**Lost to follow up or died**

29 patients

**Poor or insufficient baseline sample**28 patients

**Serum baseline analysis 130 patients**

**Suitable for analysis 290 patients**

**S4 Fig.** **ε-(γ-glutamyl)-lysine in potential TG2 substrates.** Potential TG2 substrates were incubated with TG2 for 15 hours. They were then subjected to exhaustive proteolytic digestion and the amount of ε-(γ-glutamyl)-lysine crosslink in each protein determined by amino acid analysis. NS = not statistically significant, **P* < 0.05, ****P* < 0.001. *n* = 4.

DMC, dimethyl casein; TG2, transglutaminase 2; UX, ε-(γ-glutamyl)-lysine.

**UX concentration – nmol/mg protein**

NS

**S5 Fig. Correlations of TG2 and ε-(γ-glutamyl)-lysine with proteinuria and eGFR in CKD patients.** CKD, chronic kidney disease; eGFR, estimated glomerular filtration rate; NS, not statistically significant**;** TG2, transglutaminase 2; UX, ε-(γ-glutamyl)-lysine.

**B. Human urinary Glu-Lys vs. 24h proteinuria**

1. **Human urinary TG2 vs. proteinuria**

**UX nmol/mg protein**

**TG2 concentration pg/mL**

Pearson *R* = 0.04760, NS

Pearson *R* = 0.04709, NS

**D. Human urinary TG2 vs. serum TG2 vs. CrCl**

**C. Human urinary TG2 vs. Glu-Lys**

**UX nmol/mg protein**

**Urinary TG2
concentration pg/mL**

**Serum TG2 concentration pg/mL**

**TG2 concentration pg/mL**

Pearson *R* = 0.04788, NS

Pearson *R* = 0.3796, *P* < 0.0001

**E. Human urinary TG2 vs. loss of GFR**

**eGFR decline (mL/min/1.73^2^)**

**Log_10_ TG2 concentration pg/mL**

**S6 Fig. Determination of best cut-off value for UTCR to predict CKD progression.**


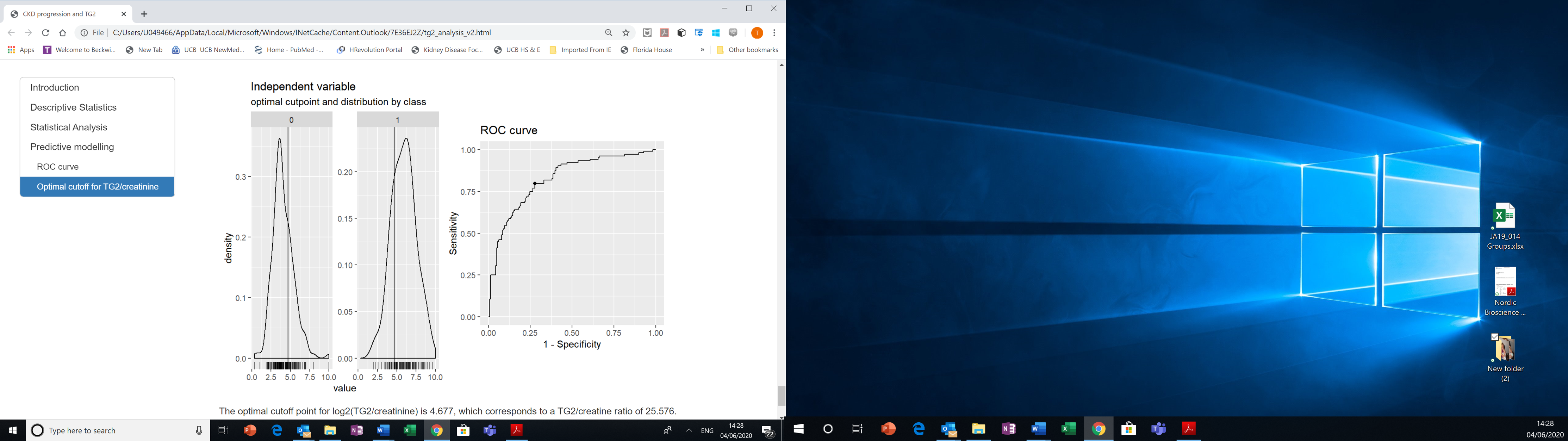
The optimal cut-off point for log_2_ (UTCR) is 4.677. This corresponds to a UTCR of 25.576. A diagnostic test using this cut-off to determine progression status (stable vs. progressor) would have a classification accuracy of 75.2%, a sensitivity of 79.8% and a specificity of 72.3%.ROC, receiver operating characteristic; UTCR, urinary TG2:creatinine ratio.

**CKD progression status**

**optimal cutpoint and distribution by class**

**ROC curve**

Stable

Progressor


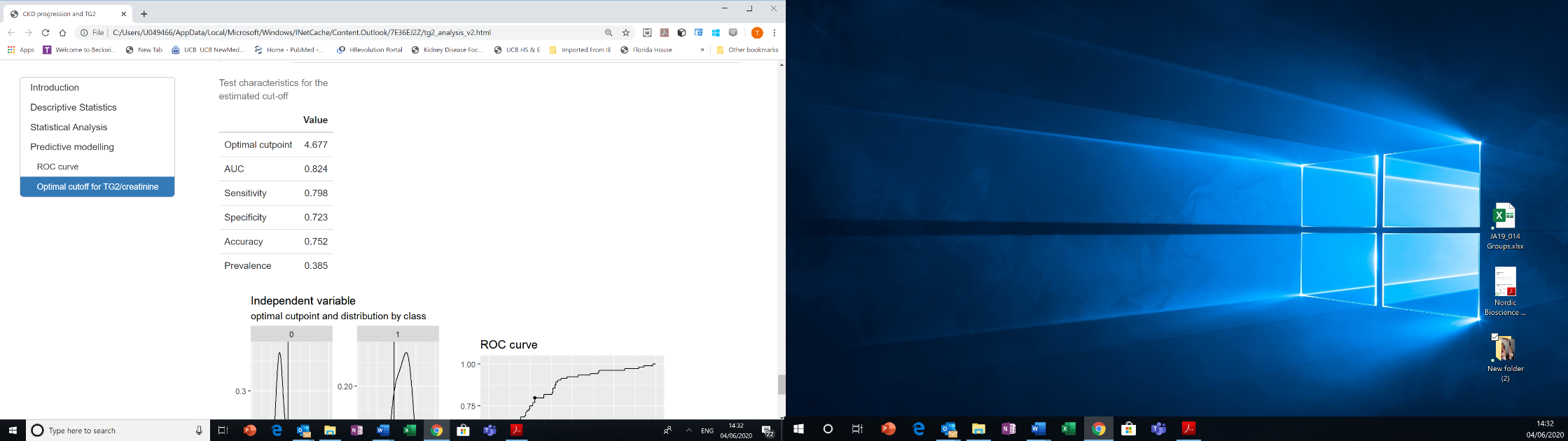


**S7 Fig. Serum TG2 ROC curve analysis.**

ROC, receiver operating characteristic; Sig., significance; Std., standard; TG2, transglutaminase 2; UACR, urinary albumin:creatinine ratio.


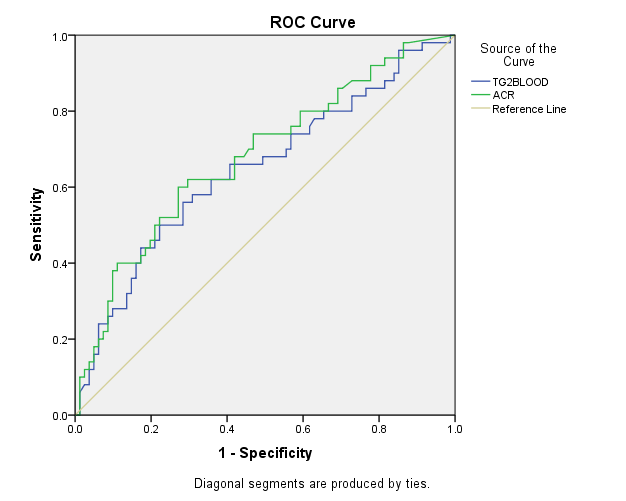


**ROC curve**

**Reference line**

**TG2 blood**

**UACR**

| **Area Under the Curve** | | | | | |
| --- | --- | --- | --- | --- | --- |
| **Test Result Variable(s)** | **Area** | **Std. Error^a^** | **Asymptotic Sig.^b^** | **Asymptotic 95% Confidence Interval** | |
|  |  |  |  | **Lower Bound** | **Upper Bound** |
| TG2 blood | 0.647 | 0.051 | 0.005 | 0.548 | 0.746 |
| UACR | 0.680 | 0.049 | 0.001 | 0.585 | 0.775 |
| The test result variable(s): TG2 blood, UACR have at least one tie between the positive actual state group and the negative actual state group. Statistics may be biased. | | | | | |
| ^a^Under the nonparametric assumption | | | | | |
| ^b^Null hypothesis: true area = 0.5 | | | | | |
